# Advances in Pathophysiology of Calcific Aortic Valve Disease Propose Novel Molecular Therapeutic Targets

**DOI:** 10.3389/fcvm.2018.00021

**Published:** 2018-03-14

**Authors:** Alexia Hulin, Alexandre Hego, Patrizio Lancellotti, Cécile Oury

**Affiliations:** ^1^GIGA Cardiovascular Sciences, Laboratory of Thrombosis and Hemostasis and Valvular Heart Disease, University of Liège, CHU Sart Tilman, Liège, Belgium; ^2^GIGA Cardiovascular Sciences, Department of Cardiology, University of Liège Hospital, Heart Valve Clinic, CHU Sart Tilman, Liège, Belgium; ^3^Gruppo Villa Maria Care and Research, Anthea Hospital, Bari, Italy

**Keywords:** calcific aortic valve disease, calcification, inflammation, oxidative stress, lipids, signal transduction

## Abstract

Calcific Aortic Valve Disease (CAVD) is the most common heart valve disease and its incidence is expected to rise with aging population. No medical treatment so far has shown slowing progression of CAVD progression. Surgery remains to this day the only way to treat it. Effective drug therapy can only be achieved through a better insight into the pathogenic mechanisms underlying CAVD. The cellular and molecular events leading to leaflets calcification are complex. Upon endothelium cell damage, oxidized LDLs trigger a proinflammatory response disrupting healthy cross-talk between valve endothelial and interstitial cells. Therefore, valve interstitial cells transform into osteoblasts and mineralize the leaflets. Studies have investigated signaling pathways driving and connecting lipid metabolism, inflammation and osteogenesis. This review draws a summary of the recent advances and discusses their exploitation as promising therapeutic targets to treat CAVD and reduce valve replacement.

## Introduction

Over the course of an average day, aortic valve (AoV) leaflets open and close 100,000 times allowing unidirectionality blood flow from the left ventricle to the systemic circulation. The proper function of AoV is achieved by thin leaflets composed of three distinct layers of extracellular matrix (ECM), rich in fibrillar collagen, glycosaminoglycans (GAGs) and elastin. Calcific Aortic Valve Disease (CAVD) appears first as AoV sclerosis developing into AoV stenosis (1, 2). Macroscopically, leaflets are thickened and progressively calcified resulting into stiff leaflets with restricted movement.

CAVD is one of the most common heart valve disease and its prevalence increases with aging (3). Nowadays, in western countries, 2.8% of the general population aged over 75 years is affected with moderate to severe aortic stenosis (3, 4). With life expectancy increasing, prevalence of heart valve disease is expecting to rise. Nevertheless, due to a lack of drug treatment (5), surgery remains the only way to treat it through surgical valve replacement or transcatheter aortic valve implantation.

The seeking of therapeutic targets relies on mechanistic understanding of CAVD. Due to its association with aging, CAVD used to be considered as a passive disease, but is now established that CAVD is an active cellular-driven regulated process (6). Heart valve homeostasis is tightly controlled by valve interstitial cells (VICs) embedded in ECM, valve endothelial cells (VECs) covering the leaflet, and circulant and resident immune cells. When CAVD develops, lipid deposition, inflammation and angiogenesis occur while VICs are entering an osteogenic program as a response to exposure to risk factors including age, congenital heart defect, male gender, tobacco use, diabetes, hypertension, obesity and dyslipidemia (7–9). As a result, homeostasis is disrupted, ECM is remodeled, and formation of calcium nodules occurs. Although mechanisms leading to CAVD are still unclear, studies on diseased human aortic valves and animal models of CAVD, reviewed by Sider *et al.* (10), have provided valuable insights into cellular components and signaling pathways involved in the pathogenesis. This review will summarize the current findings with emphasis on valuable therapeutic candidates.

### CAVD: Multi-Step Process with Endothelium Damage as Starting Point

**Figure 1:**
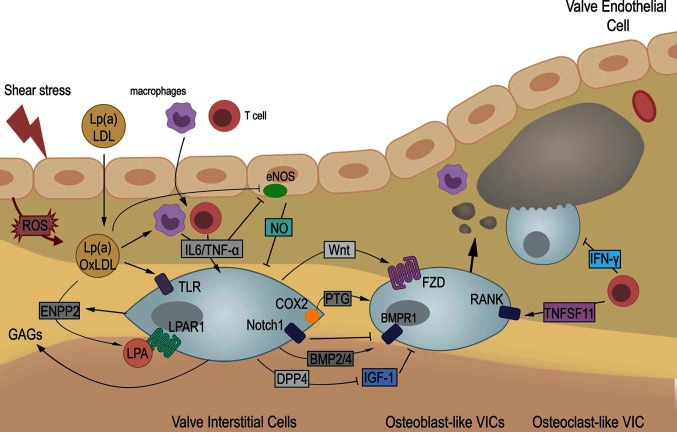
CAVD is a multi-step disease. Upon valve endothelium damage, low-density-lipoprotein (LDL) and lipoprotein a [Lp(a)] accumulate. Oxidation of LDL (oxLDL) trigger infiltration of macrophages and T cells that express pro-inflammatory cytokines among which IL-6 and TNF-α. Proinflammatory cytokines impairs protective role of valve endothelial by inhibition of *endothelial nitric oxide synthase* (*eNOS*) and production of nitric oxide (NO). Therefore, oxidative stress (ROS) increases and contributes to enhance oxLDL. Concomitantly, valve interstitial cells (VICs) get activated by cytokines and oxLDL directly, or indirectly through autotaxin (ENPP2) and lysophosphatidyl acid (LPA). Therefore, VICs enter an osteogenic differentiation leading to calcific deposit and nodule formation. Activated VICs secrete glycosaminoglycans (GAGs), favoring further accumulation of oxLDL. Increased cyclooxygenase 2 (COX2) and its product prostaglandin (PTG), Wnt and BMP signaling have been shown to drive osteogenic differentiation while inhibition of IGF-1 signaling by dipeptidyl peptidase-4 (DPP4) contributes as well to pathogenesis. Also, Notch signaling, induced by NO, repress osteogenic differentiation. Finally, T cells favor osteogenesis and osteoclast formation by production of TNFSF11 but secrete Interferon-γ (IFN-γ) which limits calcium resorption. Altogether, aortic valve leaflets gets remodeled and stiffen leading to aortic valve stenosis. ROS: Reactive Oxygen Species. LPAR1: Lysosphosphatidyl Acid Receptor 1. TLR: Toll-like receptor. IL6: interleukin 6. TNF-α: Tumor necrosis factor- α. BMP: Bone Morphogenetic Protein. Morphogen. BMPR1: BMP Receptor 1. Fzd: Frizzled. RANK: Receptor Activator of Nuclear Factor kB. TNFSF11: RANK ligand.

Endothelium dysfunction is an early feature of CAVD (11, 12) and likely the result of altered blood shear stress (13). There is indeed a spatial correlation between the calcific lesions, located almost exclusively on the aortic side of AoV leaflet, and the local hemodynamic environment (14–16).The hypothesis of hemodynamic onset is reinforced by the predisposition and accelerated progression of CAVD in patients with bicuspid aortic valve (17) that display different blood flow patterns than observed with tricuspid AoV (18, 19). Endothelium damage favors lipid deposit followed by infiltration of inflammatory cells, two hallmarks of early AoV lesions (20). Therefore, lipids and cytokines will influence neighbored VECs and VICs to promote activation of VICs, ECM remodeling and mineralization of AoV leaflets (Figure 1).

### Oxidized LDLs Mediate Inflammation and Mineralization

The importance of dyslipidemia in CAVD was confirmed by prevalence of CAVD in familial hypercholesterolemia caused by mutation of *LDL receptor (Ldlr)* and leading to abnormal circulating level of LDL (21–23). Hypercholesterolemia induced in animal models by genetic mutation (*Ldlr^−/−^, ApoE^−/−^, ApoB^100/100^*) and/or combined with enriched diet further indicate that increased lipid deposits precede the emergence of inflammatory and calcification processes (21, 24). Due to association between lipid and CAVD, clinical trial using lipid level lowering drug have been carried out, but it has shown negative results in regard with reducing CAVD (5, 25–27). One of the reasons might be that statins are ineffective to reduce Lp(a) level (28, 29) . Lp(a) consists of low density lipoprotein (LDL)-like particle in which apoliprotein(a) is covalently linked to apoliprotein B100 (30). Histopathologic studies demonstrated accumulation of apoliproteins and lipid in early stages of CAVD (31). Genome wide association study further described a SNP in *LPA* gene that was strongly associated with CAVD. Individuals with that SNP had higher Lp(a) plasma level and higher risk of aortic valve stenosis (32–34).

 Altogether, Lp(a) appears genuinely to mediate the onset of CAVD. Deciphering the pathogenic mechanisms linking Lp(a) to CAVD has been recently acknowledged as a priority (35). Several studies highlighted a link between lipid metabolism and calcification through oxidation of LDLs. Lp(a) is a carrier of oxidized phospholipids (OxPLs), used by Lp(a)-associated phospholipase A2 (Lp-PLA2), to generate lysophosphatidyl choline (LPC), all highly expressed in human CAVD (36, 37). LPC is then transformed into lysophosphatidyl acid (LPA) by ectonucleotide pyrophosphatase/phosphodiesterase 2 (ENPP2), secreted by stimulated VICs (38). LPA is also produced during non- oxidative transformation of LDLs. Therefore, LPA activates VICs through enzymatic LPAR1/RhoA/NF-κb signaling, and mediates mineralization through *BMP2* expression (38, 39). The requirement for RhoA to promote calcific nodule was also illustrated *in vitro* (40). The signaling pathway is confirmed with decreased AoV mineralization when using Ki16425, an inhibitor of LPAR1, in *Ldlr^−/−^, ApoB^100/100^* mice fed with high fat and high sucrose diet (39). It is important to mention that changes in the ECM, with accumulation of glycosaminoglycans, precede and favor oxLDL retention (24, 41, 42).

The findings indicate that lowering Lp(a), OxLDL or targeting LPAR1 are attractive options and might be used to prevent the onset of CAVD. Multiple treatment options are currently suggested to decrease Lp(a). IONIS-APO(a)_Rx,_ and IONIS-APO(a)-L_Rx_, antisense oligonucleotide targeting *Apo(a)* mRNA have been shown to lower Lp(a) level (43). Targeting Proprotein Convertase Subtilisin/Kexin type 9 (PCSK9), a hepatic protease that promotes LDLR destruction, might be a way to decrease LDL and oxidative products. This might be achieved with monoclonal antibodies, Alirocumab and Evolocumab (44), or by using Inclisiran, a small RNAi targeting *PCSK9* (45, 46).

### Inflammation Contributes to Calcification

Inflammation occurs after endothelium activation and lipid deposition. Microarray analysis of human CAVD (47) and Rapacz familial hypercholesterolemia swine, an established model of human FH (21) shows upregulation of inflammation-related genes and chemokines. Histological studies present inflammatory cells, composed of macrophages, B and T cells found near osteoblast-like cells and calcified area in human CAVD (20, 48, 49). PET imaging using 18-Flurodexoxyglucose uptake (18F-FDG) to monitor inflammation reports higher 18F-FDG uptake in patients with AoV sclerosis and stenosis and a raise of the activity as the disease gets more severe (50).

Besides activation of endothelial cells (11, 12), OxLDLs trigger proinflammatory cytokines expression and promotes infiltration of immune cells into AoV leaflets (42, 51, 52). In diseased AoV, higher oxLDL content correlates with higher amounts of inflammatory cells (53) . During inflammation, immune cells secrete inflammatory cytokines including IL-2 (54), IL-1β (55), TNF-α (56, 57) , IL6(58) and MMPs (55, 59) than stimulates VICs, ECM remodeling and promote the expression of genes involved in osteogenesis (52). Altogether, data support that CAVD is an inflammatory disease, and inflammation may drive calcification.

Although inflammation precedes ECM remodeling and calcification, inflammation over the course of the disease has not been fully explored yet. Similarly, immune cells display a broad heterogeneity with specific function. Thorough characterization of macrophages, T cells or B cells is now just starting to be done in the context of CAVD. M1 macrophage subset have recently be found to be the predominant macrophage subset in CAVD, promoting osteogenic differentiation of VICs through TNF-α and IL-6 secretion (58, 60). T cells are also reported surrounding calcified area. T cells favor calcification through cytokine TNF-α and TNFSF11 expression (56, 61, 62). Increased T cells in diseased AoV is likely the result of increased circulating CD8 +T cells (63). Activated T cells infiltrate the leaflets and surround calcified area and display high level of inflammatory cytokine IFN-γ (62). Although TNFSF11 promotes osteoclast activity, aberrant IFN-γ level impairs calcium resorption by valve osteoclast. Therefore, calcium accumulates in the leaflets and facilitates nodule formations (62). A similar study indicates that macrophages surrounding calcium deposits in human atherosclerotic are defective and unable to resorb calcification (64). Such role of macrophage in CAVD have not been explored yet. Circulating Tregs are also measured in patients with CAVD and associate with disease progression (65). Although dendritic cells are found abundantly in heart valve and accumulate in AoV stenosis, their contribution to CAVD is still unknown (51, 66).

Deeper understanding of regulation, timing and functional role of immune cells in CAVD will bring valuable information to determine how targeting inflammation might help preventing pathogenesis.

### VECs Are Natural Inhibitors of Calcification, Through NO Release, but Activators Through Oxidative Stress

Inflammatory cytokines, TNF-α and IL-6, induce valve endothelial-to-mesenchymal (EMT) transformation through Akt/NF-κb signaling and reduce *endothelial nitric-oxide synthase* (*eNOS*) expression (67). Although some markers of EMT are measured in human calcified aortic valves (67), studies have still to address if EMT contribute to pathogenesis of CAVD.

VECs have the particularity to display side-specific heterogeneity. Endothelium on the aortic side displays an antioxidative and anti-inflammatory phenotype defined by its RNA expression profile (15). Thus, aortic side of AoV demonstrates protection against repetitive insult in normal AoV. As consequence, VECs are releasing nitric-oxide (NO), a natural inhibitor of pathogenic differentiation of VICs into myofibroblast andosteoblasts (68). Increased NO release has been shown to inhibit calcific nodule formation *in vitro* (69) and *in vivo* with atorvastatin treatment (70). On the opposite, in CAVD, altered mechanical stimulus, oxLDLs or TNF-α impair *eNOS* expression (68, 71, 72). Concomitantly, uncoupling of NO synthase leads to increased production of superoxide and oxidative stress which drives calcification (73).The critical role of endothelium and *eNOS* was further illustrated through modulation of a multifunctional enzyme dipeptidyl peptidase-4 (DPP4) and insulin growth factor-1 (IGF-1). Upon NO depletion, DPP4 increases in human VICs and limits IGF-1 signaling leading to enhanced calcification. Treatment of rabbit and mouse model of CAVD with Sitagliptin, a selective DPP4 inhibitor, was protective against AoV calcification (74). Similarly, the protective role of VECs is illustrated by TGF-β1 expression that translocates Sox9 into VICs nucleus and prevent calcific nodule formation (75, 76). Therefore, enhancing protective role of VECs, during early phase of disease, must be exploited. Notably, increasing NO production with statins or using DPP4 inhibitor ,broadly used as hypoglycemic drugs for treatment of type 2 diabetes mellitus, might mitigate CAVD.

### VICs Differentiate Into Osteoblast-Like Cells and Mineralize the Leaflets

Histological studies report the formation of bone nodules in stenotic CAVD resulting from deposition of calcium in the form of hydroxyapatite in the valve leaflet (49). Once heart valve development is complete, VICs become quiescent, but in disease get activated and turn into active phenotype. In response to pathological stimuli, VICs differentiate into osteoblast-like cells with abnormal expression of typical bone genes, including *Runx2, Alkaline Phosphatase (ALP), Osteopontin (SPP1)*, *Osteocalcin* (*BGLAP*) (47) resulting in calcified ECM. Apart from promoting inflammation, OxLDLs and Lp(a) can also directly activate VICs through LPAR1 (38, 39) and TLR activation (52, 77–79), This interaction contributes to trigger GAG accumulation, in a positive feedback loop, and upregulates osteogenic gene expression through *BMP2* and *IL6* expression (38, 42, 80).

Different molecular mechanisms are involved in VICs osteogenic differentiation and shared with bone formation (81, 82). Stimulation of VICs culture with OxLDLs and hypercholesterolemia animal model have been used to investigate signaling pathway underlying osteogenic differentiation. Also studies in *klotho* null mice have been useful to investigate AoV calcification with minimal inflammation (83).*BMP2*, along with osteogenic gene expression, are the usual markers measured to assess VICs osteogenic differentiation. BMP signaling is increased in human CAVD illustrated by increased BMP2, BMP4 ligands and phosphorylation of Smad1/5/8 (82, 84, 85). Downregulation of* Smad6*, an inhibitor of BMP signaling, enhance BMP signaling (84, 86). Inhibition of osteogenic gene expression and calcific nodule formation by targeting Alk3, BMP receptor type-1A, strongly indicate that LDN-193189, a small molecule inhibitor of BMP signaling, should be used to prevent calcification in late stage of CAVD (85).

Mutation in *Notch1* and its association with BAV and AoV calcification highlighted the role of Notch signaling in CAVD (87). Later, studies confirms that Notch signaling represses osteogenic gene expression (88, 89) and is regulated by NO released by endothelial cells (90). Decreased Notch signaling is not just observed in patients with mutated* Notch1* but also in patients with idiopathic CAVD where increased long non-coding RNA *H19,* resulting from hypomethylation, prevents *Notch1* expression (91).The role of prostaglandins has been illustrated in osteogenesis (92, 93), but only recently in CAVD. Prostaglandins are synthesized by COX2, an enzyme highly expressed by VICs in CAVD (94). Pharmacological inhibition of COX2 activity with Celecoxib, a nonsteroidal anti-inflammatory (NSAID) drugs, is sufficient to reduces AoV calcification in *Klotho* null mice (94). Celecoxib is clinically used to treat joint and/or muscle pain(95) but was associated with increased cardiovascular risk (96). Cardiovascular safety of celecoxib is nowadays controversial (97) as recent report indicate that cardiovascular risk associated with moderate doses of celecoxib is not greater than associated with non-selective-NSAID ibuprofen (98). Additional research must evaluate the effectiveness of COX2 inhibitor in human CAVD.

Non-canonical Wnt5b and Wnt11 ligands are found elevated in macrophages of human calcified AoV. Moreover, the ligands stimulate VICs, apoptosis and calcium deposits (99). Abundant expression of Fzd receptors and co-receptors Lrp5/6 also suggest the involvement of canonical Wnt/β-catenin signaling in CAVD (81, 100). *In vitro*, Wnt treatment of VICs inhibit chondrogenic differentiation and promote osteogenic gene expression (101, 102) while Lrp5/6 is required to promote calcification in hypercholesterolemia mouse model (103). In *Axin2* KO mice, increased canonical Wnt/β-catenin signaling promotes ECM remodeling and BMP signaling but fails to calcify AoV (104). The findings illustrate that Wnt signaling is required but might not be sufficient to promote end-stage calcification. These data illustrate the importance to further study the role of Wnt signaling in CAVD as specific inhibitors are being tested (105).

VIC osteogenic differentiation has been one of the most studied process in CAVD due to available cell culture model. However, VIC remains a poorly defined cell type. Heterogeneity of VIC population is underappreciated during heart valve homeostasis and disease. Being able to define which cell type is activated and/or differentiated across disease is a major goal in order to present innovative therapeutic options.

## Conclusions

CAVD is a complex multi-step event that involves numerous biological processes from lipid accumulation, inflammation to osteogenesis. Understanding the underlying molecular and cellular processes is crucial in the establishment of therapeutic targets. Clinical, histological and animal model studies have allowed better characterization of the disease and show the importance of cross-talk between lipids, immune cells, VECs and VICs. As a result, putative molecular targets with available treatments (Table 1) emerge for each stage of CAVD. Giving the multifactorial and complex interplay, timing and combination of therapy should be considered. In the context of appropriate therapeutic timing, accurate biomarkers should be defined. Similarly, thorough knowledge of the heterogeneity and function of valve cell subtype, over the course of the disease, may provide better targeting of the “diseased” cells. Overall, recent advances and future directions bring hope for the development of efficient drug treatment and for the reduction of valve replacement surgeries.

**Table 1:** Putative available therapeutic treatments and molecular targets that might affect the pathophysiology of CAVD. In brackets, species where the drug effect has been reported.

**Putative Therapeutic treatments**	**Molecular Targets**	**Biological process**
IONIS-APO(a)_Rx_IONIS-APO(a)-L_Rx_	*Apo(a)*	Lp(a) level lowering (human)
AlirocumabEvolocumabInclisiran	PCSK9	Lipid lowering (human)
Statins	HMG-CoA reductase	Lipid lowering (human)Promotes NO release/inhibition of calcification (rabbit)
Ki16425	LPAR1	Inhibition of calcification (mouse)
Sitagliptin	DPP4	Inhibition of calcification (mouse)
LDN-193189	BMPR1A	Inhibition of calcification (mouse)
Celecoxib	COX2	Inhibition of calcification (mouse)

## Author Contributions

AHu wrote the manuscript. AHe drafted the figure. PL provided intellectual contributions and edited the manuscript. CO drafted and revised the manuscript.

## Conflict of Interest Statement

The authors declare that the research was conducted in the absence of any commercial or financial relationships that could be construed as a potential conflict of interest.
